# Effects of Tranexamic Acid in Combination with Teicoplanin Against *Staphylococcus isolates*: Results from an In Vitro Study

**DOI:** 10.3390/ijms27135764

**Published:** 2026-06-26

**Authors:** Yasin Koker, Sahika Cingir Koker, Irem Dogan Turacli, Mahmut Nedim Sultan, Burak Akan, Berk Guclu

**Affiliations:** 1 Department of Orthopedics and Traumatology, Faculty of Medicine, Ufuk University, Ankara 06520, Türkiye; gucluberk@yahoo.com; 2 Department of Medical Biology, Faculty of Medicine, Ufuk University, Ankara 06520, Türkiye; sahika.koker@gmail.com (S.C.K.); doganirem@gmail.com (I.D.T.); 3 Department of Microbiology, Faculty of Medicine, Ufuk University, Ankara 06520, Türkiye; nedimsul@yahoo.com; 4 Department of Orthopedics and Traumatology, Acibadem Atasehir Hospital, Istanbul 34758, Türkiye; burakakan1977@yahoo.co.uk

**Keywords:** *Staphylococcus epidermidis*, tranexamic acid, teicoplanin, biofilm, periprosthetic joint infection, orthopedic infection

## Abstract

*Staphylococcus epidermidis* is a major cause of periprosthetic and other implant-associated orthopedic infections because of its ability to adhere to biomaterial surfaces and form biofilm. Tranexamic acid (TXA) is routinely used in arthroplasty to reduce perioperative blood loss; however, emerging evidence suggests that it may also modulate bacterial behavior and antibiotic activity. This study investigated the in vitro effects of TXA in combination with teicoplanin on planktonic growth and biofilm biomass formation in clinical *Staphylococcal isolates.* Clinical Staphylococcal isolates were evaluated using disk diffusion assays, microtiter plate-based planktonic growth assays, and crystal violet biofilm biomass assays. Microplate-based growth and biofilm assays were performed using five clinical isolates, whereas disk diffusion assays were performed using a separate set of seven clinical staphylococcal isolates. Teicoplanin was tested at literature-based low concentrations of 0.1 and 0.4 µg/mL, either alone or in combination with TXA at 10 and 50 mg/mL. In disk diffusion assays, inhibition zone diameters were quantified using ImageJ. Planktonic growth was assessed by optical density at 600 nm, and biofilm biomass accumulation was quantified by crystal violet staining at 570 nm. Disk diffusion data were analyzed using paired t-tests, while microplate-based growth and biofilm data were analyzed using two-way analysis of variance (ANOVA) followed by Tukey’s multiple-comparisons test. In disk diffusion assays, TXA co-application was associated with larger teicoplanin inhibition zones on both blood agar and Mueller–Hinton agar, suggesting an increased apparent inhibitory zone under agar-based conditions. In microplate-based planktonic growth assays, responses were isolate-dependent. However, co-exposure to TXA, particularly at 50 mg/mL, was associated with reduced OD600-based bacterial growth in several isolates compared with teicoplanin alone. A similar isolate-dependent pattern was observed for crystal violet-based biofilm biomass accumulation. In most tested isolates, teicoplanin combined with 50 mg/mL TXA was associated with lower biofilm biomass than teicoplanin alone, whereas one isolate showed minimal responsiveness. Under the tested in vitro conditions, TXA–teicoplanin co-exposure was associated with reduced planktonic growth and crystal violet-based biofilm biomass accumulation in several clinical staphylococcal isolates. However, because TXA-only controls were not available across the full experimental framework and formal synergy assays were not included, these findings do not establish synergistic activity or distinguish combination-specific effects from TXA-associated effects alone. Further studies are needed to clarify the biological and translational relevance of these observations.

## 1. Introduction

Periprosthetic joint infections (PJI) remain one of the most devastating complications of arthroplasty despite the overall success of modern joint replacement surgery [1,2,3]. They are most frequently caused by staphylococci, with *Staphylococcus aureus* and coagulase-negative staphylococci [4], particularly *Staphylococcus epidermidis*, representing the dominant pathogens in most series [5]. This microbiological profile is clinically important because the prognosis of PJI is often unfavorable, requiring complex revision strategies, prolonged antibiotic exposure, and repeated follow-up, while recurrence, functional impairment, and even mortality remain important concerns [6,7].

Among the causative microorganisms, *S. epidermidis* is of particular relevance because it represents both a common skin commensal and an opportunistic pathogen in device-associated infections [8,9,10]. Under normal conditions, *S. epidermidis* is an important member of the human skin microbiota and may contribute to skin barrier homeostasis, immune education, and colonization resistance against potentially pathogenic microorganisms, including *Staphylococcus aureus* [8,11,12]. Therefore, it should not be regarded solely as a harmful bacterium. However, when introduced into normally sterile sites or when biomaterial surfaces are present, *S. epidermidis* can become clinically relevant despite its relatively low intrinsic virulence [8,9,10]. In orthopedic implant-associated infections, this clinical relevance is largely attributable to its ability to adhere to prosthetic materials and persist on implant surfaces, particularly in chronic and low-grade infections [10,13,14].

The pathogenic behavior of *S. epidermidis* in device-associated settings is closely linked to its ability to adhere to biomaterials and form biofilm [14,15,16]. Once established, biofilm substantially reduces susceptibility to host immune clearance and antimicrobial treatment, thereby contributing to diagnostic difficulty, treatment failure, and recurrence [17,18,19]. This biofilm-driven behavior explains why *S. epidermidis* remains clinically relevant in orthopedic implant-associated infections despite its beneficial commensal role on healthy skin and its relatively low intrinsic virulence [8,9,14,15]. Accordingly, interventions targeting bacterial growth and biofilm biomass accumulation may be relevant in orthopedic surgery [14,15].

Tranexamic acid (TXA), an antifibrinolytic agent widely used in arthroplasty and other major orthopedic procedures, is routinely administered to reduce perioperative blood loss and transfusion requirements [20]. Beyond its established hemostatic role, emerging experimental evidence suggests that TXA may also influence staphylococcal growth and antibiotic activity under certain conditions [21,22,23]. In vitro studies suggest that TXA may reduce planktonic bacterial growth, whereas its effects on biofilm are less consistent; moreover, TXA has been reported to modify the activity of conventional anti-staphylococcal antibiotics under some experimental conditions [21,22,24]. Since reducing perioperative blood loss and preventing infection are both important goals in orthopedic surgery [20,25], and infection prevention remains a major goal in arthroplasty [1], the concurrent perioperative use of TXA and teicoplanin may be of practical clinical relevance.

Based on this, the present study aimed to evaluate the in vitro effects of TXA in combination with teicoplanin on selected clinical staphylococcal isolates. Specifically, we examined whether the addition of TXA altered bacterial growth and biofilm biomass accumulation compared with teicoplanin alone under the experimental conditions.

## 2. Results

**Figure 1:**
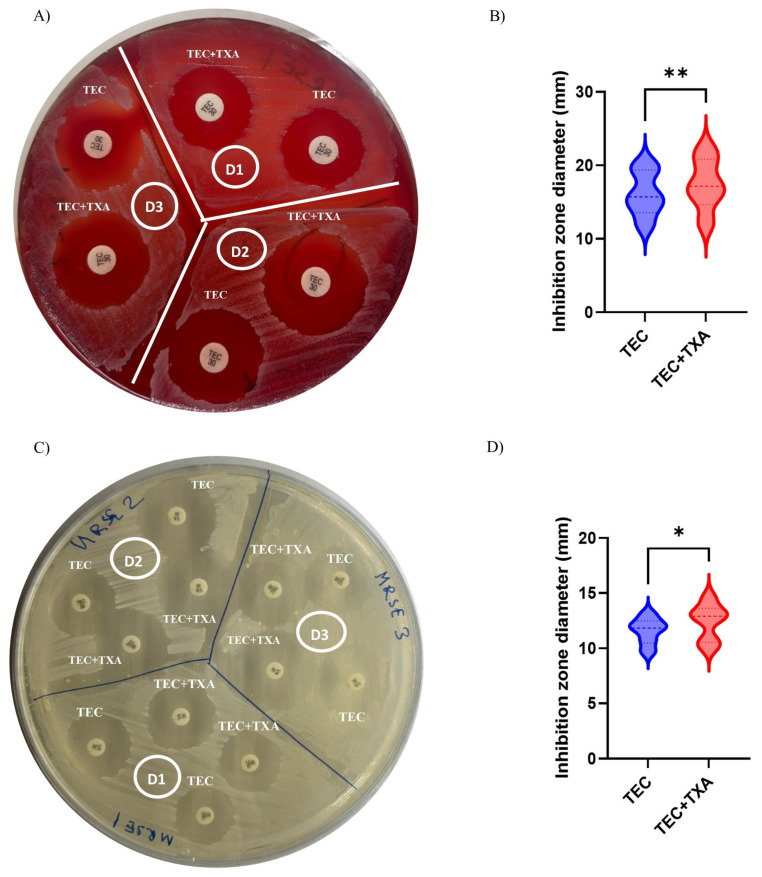
Representative disk diffusion images and quantification of inhibition zone diameters for teicoplanin (TEC) alone or in combination with TXA. (**A**) Representative blood agar plate showing inhibition zones around TEC disks in the absence or presence of TXA with three different MRSE isolates. (**B**) Quantification of inhibition zone diameters measured from different blood agar plate images; in total from eight different isolates. (**C**) Representative standard agar plate showing inhibition zones around TEC disks in the absence or presence of TXA. (**D**) Quantification of inhibition zone diameters measured from different standard agar plate images; in total from seven different isolates. Inhibition zone diameters were quantified in millimeters (mm) using ImageJ after scale calibration based on disk diameter. Data are shown as individual values with distribution. Statistical analysis was performed using a paired t-test. * *p* < 0.05, ** *p* < 0.01.

To first assess whether TXA modulates the antibacterial activity of teicoplanin at the phenotypic level, disk diffusion assays were performed on both blood and standard agar plates by using seven different isolates (Table 1). Representative images showed that the addition of TXA increased the inhibition zone diameter around teicoplanin disks compared with teicoplanin alone. This effect was observed in both media, with significant increase in inhibition zone diameter following ImageJ-based quantification (Figure 1A–D). These findings suggest that TXA–teicoplanin co-exposure was associated with larger inhibition zones under the tested conditions.

**Figure 2:**
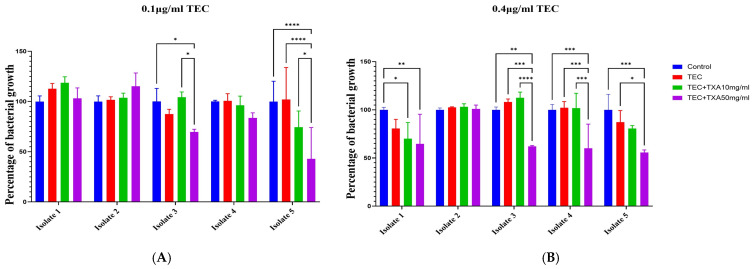
Bacterial growth responses of five clinical staphylococcal isolates to teicoplanin and TXA. (**A**) Growth of five clinical isolates after treatment with 0.1 µg/mL teicoplanin alone or in combination with TXA (10 mg/mL or 50 mg/mL). (**B**) Growth of five clinical isolates after treatment with 0.4 µg/mL teicoplanin alone or in combination with TXA (10 mg/mL or 50 mg/mL). Untreated controls were included for comparison. Data are expressed as percentage of bacterial growth relative to untreated controls and shown as mean ± SD with individual replicate values overlaid. Statistical significance between the indicated groups was determined by two-way ANOVA with multiple comparisons. Exact mean ± SD values for the 0.1 and 0.4 µg/mL teicoplanin treatment groups are provided in Supplementary Tables S1 and S2, respectively. * *p* < 0.05, ** *p* < 0.01, *** *p* < 0.001, **** *p* < 0.0001.

We next examined bacterial growth in five clinical *Staphylococcal* isolates (Table 1) different than the ones used in disk diffusion assays: treated with teicoplanin alone or in combination with TXA at two concentrations (10 and 50 mg/mL). In isolate 1, 0.1 µg/mL teicoplanin had no clear effect on bacterial growth, either alone or in combination with TXA. At 0.4 µg/mL teicoplanin, however, the addition of TXA at both 10 and 50 mg/mL was associated with a significant reduction in bacterial growth. No significant treatment-related change in bacterial growth was observed in isolate 2. In isolate 3, teicoplanin alone at either 0.1 or 0.4 µg/mL showed limited effect, whereas the addition of 50 mg/mL TXA was associated with reduced bacterial growth. In isolate 4, 50 mg/mL TXA showed a trend toward reduced bacterial growth in the presence of 0.1 µg/mL teicoplanin, and this effect reached statistical significance at 0.4 µg/mL teicoplanin. In isolate 5, the addition of 50 mg/mL TXA was associated with reduced bacterial growth at both teicoplanin concentrations (Figure 2A,B).

**Figure 3:**
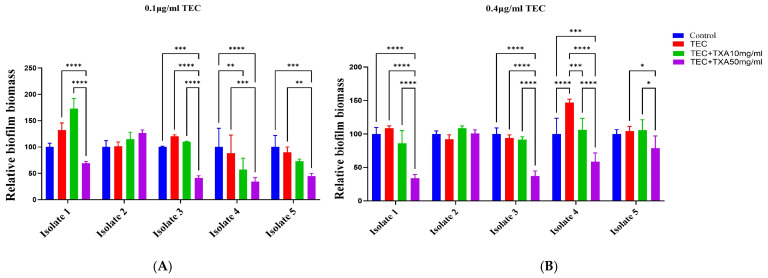
Biofilm biomass responses of five clinical staphylococcal isolates to teicoplanin and TXA. (**A**) Relative biofilm biomass of five clinical isolates following treatment with 0.1 µg/mL teicoplanin alone or in combination with TXA (10 mg/mL or 50 mg/mL). (**B**) Relative biofilm biomass of five clinical isolates following treatment with 0.4 µg/mL teicoplanin alone or in combination with TXA (10 mg/mL or 50 mg/mL). Untreated controls were included for comparison. Biofilm biomass was assessed by crystal violet staining and is expressed relative to untreated controls. Data are shown as mean ± SD with individual replicate values overlaid. Statistical significance between the indicated groups was determined by two-way ANOVA with multiple comparisons. Exact mean ± SD values for the 0.1 and 0.4 µg/mL teicoplanin biofilm biomass groups are provided in Supplementary Tables S3 and S4, respectively. * *p* < 0.05, ** *p* < 0.01, *** *p* < 0.001, **** *p* < 0.0001.

We next evaluated biofilm biomass accumulation following treatment with teicoplanin alone or in combination with TXA. In isolate 1, teicoplanin alone did not clearly reduce biofilm biomass accumulation, whereas the addition of 50 mg/mL TXA significantly reduced biofilm biomass at both 0.1 and 0.4 µg/mL teicoplanin. A similar pattern was observed in isolates 3, 4, and 5. In contrast, isolate 2 showed limited response under the tested conditions. Overall, the combination of teicoplanin and 50 mg/mL TXA was associated with reduced biofilm biomass accumulation in most tested isolates (Figure 3A,B and Supplementary Figure S1).

## 3. Discussion

Biofilm-associated infections remain a major challenge in orthopedic and implant-related settings because bacterial attachment to biomaterial surfaces can promote persistence and reduce antimicrobial susceptibility [14,18]. TXA is commonly used in arthroplasty to limit perioperative blood loss [20], but recent experimental evidence suggests that it may also influence bacterial growth [21], biofilm formation [24], and antibiotic activity under selected conditions [22]. In this study, we examined the in vitro effects of TXA combined with teicoplanin on planktonic growth and crystal violet-based biofilm biomass formation in clinical *S. epidermidis* isolates.

For the microplate-based planktonic growth and biofilm biomass assays, five clinical isolates were used, including two blood isolates, two wound swab isolates, and one urine isolate. The inclusion of isolates from different clinical sources may be considered a strength because it introduces a degree of clinical heterogeneity into the isolate panel. Isolates originating from different anatomical or clinical contexts may differ in growth characteristics, antimicrobial susceptibility, and biofilm-associated behavior [15,16,18]. Such variability is relevant when exploring isolate-dependent responses in vitro.

Overall, the observed responses varied across isolates and treatment conditions. In several isolates, particularly under 50 mg/mL TXA co-exposure, TXA combined with teicoplanin was associated with lower OD600-based bacterial growth and reduced crystal violet-based biofilm biomass compared with teicoplanin alone. However, isolate 2 showed limited responsiveness, supporting the idea that the effect of TXA–teicoplanin co-exposure may be isolate-dependent. This variability may reflect differences in baseline susceptibility, prior antimicrobial exposure, biofilm-forming capacity, or genetic and regulatory features of the isolates, although these parameters were not directly examined in the present study. Importantly, although additional TXA-only control experiments were performed in representative isolates and provided in Supplementary Figure S2, TXA-only controls were not available across the full experimental framework, and MIC/FICI and time-kill assays were not performed. Therefore, the present study cannot definitively distinguish TXA-specific effects from a true TXA–teicoplanin interaction, and the findings should be interpreted cautiously within the limits of the current in vitro design.

Disk diffusion assays performed on blood agar and Mueller–Hinton agar provided supportive agar-based phenotypic observations. Representative images showed larger inhibition zones around teicoplanin disks in the presence of TXA, and ImageJ-based quantification showed increased inhibition zone diameters under the tested agar conditions. However, these findings should be interpreted cautiously. The disk diffusion assays were performed using a separate set of seven clinical staphylococcal isolates, whereas the microplate-based planktonic growth and crystal violet biofilm assays were performed using five clinical isolates. Therefore, the disk diffusion data should be considered supportive phenotypic observations rather than isolate-matched validation of the microplate-based findings. In addition, inhibition zone diameter may be influenced not only by antimicrobial activity but also by agar composition, compound diffusion, and local physicochemical interactions between TXA and teicoplanin.

The crystal violet assay showed that TXA–teicoplanin co-exposure was associated with lower biofilm biomass accumulation in most tested isolates, especially at 50 mg/mL TXA. These observations may be relevant in the context of implant-associated staphylococcal infections, where early bacterial attachment and biofilm development contribute to persistence. However, crystal violet staining measures total biofilm biomass rather than viable biofilm-associated bacteria. It also does not provide information on biofilm architecture, matrix composition, or the spatial organization of biofilm-associated cells. Therefore, the present findings should not be interpreted as evidence of biofilm eradication or bactericidal activity against biofilm-embedded bacteria. Rather, they indicate that TXA–teicoplanin co-exposure was associated with reduced biofilm biomass accumulation under the tested in vitro conditions.

The mechanisms by which TXA may influence bacterial growth or biofilm-associated responses remain uncertain. Several non-mutually exclusive explanations may be considered. Previous studies have shown that TXA can alter staphylococcal growth dynamics and modify the activity of anti-staphylococcal antibiotics under selected experimental conditions [21,22]. In addition, because staphylococcal biofilm formation and antimicrobial susceptibility are influenced by surface attachment, cell surface-associated factors, extracellular matrix organization [16], and environmental physicochemical conditions [26], TXA may indirectly affect bacterial growth or antibiotic susceptibility through these processes. However, the present study did not include mechanistic assays evaluating membrane integrity, cell wall alterations, antibiotic penetration, quorum sensing, gene expression, or biofilm matrix composition. Therefore, these possible mechanisms should be regarded as hypotheses rather than confirmed explanations.

The TXA concentrations used in this study should be interpreted in the context of local or topical exposure rather than systemic plasma levels. The 10 mg/mL concentration was selected because it has been used in previous TXA–antibiotic combination studies and described as relevant for local TXA exposure [22], whereas 50 mg/mL has been evaluated in experimental implant-associated infection and biofilm models [24]. Nevertheless, the present study did not measure local TXA pharmacokinetics, and the clinical relevance of these concentrations may vary depending on the route of administration, dose, dilution volume, drainage, and exposure time in orthopedic surgery. Future studies should evaluate local TXA concentrations after orthopedic administration and test broader dose ranges. In addition, host cell cytotoxicity was not evaluated in the present study. Therefore, the safety of these TXA concentrations for mammalian cells cannot be inferred from the current bacterial assays. Future studies should include host cell viability assays, such as MTT or comparable cytotoxicity assays, together with local pharmacokinetic assessment to better evaluate the translational relevance and safety of these concentrations.

Previous studies have mainly examined TXA in relation to vancomycin or gentamicin, rather than teicoplanin. Benjumea et al. reported that TXA combined with vancomycin or gentamicin showed synergistic activity against selected staphylococcal strains in vitro, although the effect varied among strains [22]. A later validation study also supported the antibacterial effect of topically applied TXA and confirmed that the synergistic interaction with vancomycin or gentamicin occurred only in selected strains [23]. Wang et al. further reported that TXA may protect against implant-associated infection by reducing biofilm formation in an experimental model [24]. These findings are broadly consistent with our observation that TXA–teicoplanin co-exposure was associated with reduced planktonic growth and crystal violet-based biofilm biomass in selected isolates. However, unlike prior vancomycin-focused studies, the present study did not include TXA-only controls, checkerboard/FICI analysis, or time-kill experiments. Therefore, our findings should be interpreted more cautiously as preliminary teicoplanin-based observations rather than definitive evidence of synergy.

Although the present assays provide useful phenotypic information, they do not allow definitive assessment of antimicrobial synergy. Disk diffusion assays may reflect changes in inhibition zone diameter, OD600 measurements provide an indirect estimate of bacterial growth, and crystal violet staining reflects total biofilm biomass. However, these approaches cannot distinguish synergistic, additive, indifferent, or bactericidal interactions. This is particularly important because TXA-only controls were not available across the full experimental framework. Therefore, the current design cannot determine whether the observed responses reflect a specific TXA–teicoplanin interaction, an additive effect, or TXA-driven activity alone. Accordingly, the present findings should be interpreted as combination-associated effects under the tested in vitro conditions rather than definitive evidence of potentiation or synergy. Future studies should include TXA-only controls, MIC testing, checkerboard/FICI analysis, and time-kill assays to better define the nature of the interaction between TXA and teicoplanin.

This study has several limitations. First, as an in vitro study, it cannot fully reflect the complexity of the in vivo environment, including host immune responses, tissue penetration, and pharmacokinetic factors. Second, the number of isolates was limited. Five clinical isolates were used for the microplate-based planktonic growth and crystal violet biofilm biomass assays, while disk diffusion assays were performed using a separate set of seven clinical staphylococcal isolates. Because these assays were not performed on the same isolate panel, direct isolate-matched comparison between agar-based inhibition and microplate-based growth or biofilm responses was not possible. This is particularly relevant because the responses varied among isolates. Therefore, the findings should be considered preliminary and limited to the tested isolate sets.

The use of single-species staphylococcal biofilm models is another limitation of this study. Clinical biofilm-associated infections may involve polymicrobial communities, including interactions with organisms such as *Pseudomonas aeruginosa* [27,28,29]. Future studies should therefore evaluate TXA–teicoplanin effects in polymicrobial biofilm models to better reflect complex infection settings.

Another limitation is related to the readouts used in this study. OD600 measurements provide only an indirect estimate of bacterial growth and do not directly quantify viable cells or distinguish live from dead bacteria. Therefore, OD600-based changes should not be interpreted as direct evidence of bactericidal activity or reduced viable bacterial burden. To partially address this limitation, additional CFU-based viability assays were performed in two representative isolates under selected key conditions. The Supplementary Data (Supplementary Figure S3) were broadly consistent with the OD600-based pattern, particularly for the TXA 50 mg/mL + teicoplanin 0.4 µg/mL condition, but they remain limited to representative isolates rather than the full isolate panel. Future studies should include CFU counting and/or viability assays to confirm whether the observed changes correspond to reductions in viable bacterial counts. Similarly, crystal violet staining measures total biofilm biomass, but it cannot distinguish viable from non-viable biofilm-associated bacteria or provide information about biofilm architecture or matrix composition. In addition, treatments were applied at the time of inoculation, so this study evaluated bacterial growth and biofilm development rather than eradication of established biofilms.

In addition, the experiments were performed using technical replicates, while independent repeat experiments for the same isolate were not included. Although the isolate panel provided biological heterogeneity by including independent clinical specimens, future studies incorporating additional independent biological replication at the isolate level would further strengthen reproducibility.

Finally, disk diffusion, OD600 measurement, and crystal violet staining are not sufficient to define antimicrobial synergy. Because checkerboard/FICI analysis and time-kill assays were not performed, we cannot determine whether the observed responses represent synergy, additivity, indifference, or TXA-driven effects. Future studies should evaluate larger and better-characterized isolate panels using MIC testing, checkerboard/FICI analysis, time-kill assays, CFU-based viability assays, and mature biofilm models.

## 4. Materials and Methods

### 4.1. Bacterial Isolates

In this study, clinical staphylococcal isolates obtained from different clinical specimens were used. Each isolate represented an independent clinical specimen. For each isolate and treatment condition, assays were performed with 3–5 technical replicates depending on the experimental setup. For the microplate-based planktonic growth and crystal violet biofilm biomass assays, five clinical staphylococcal isolates were examined, including two methicillin-resistant *S. epidermidis* (MRSE) isolates and three coagulase-negative staphylococcal isolates. These five isolates were recovered from two blood samples, two wound swabs, and one urine sample (Table 1). In addition, disk diffusion assays were performed using a separate set of clinical staphylococcal isolates, as summarized in Table 1. Fresh bacterial cultures were prepared by subculturing the isolates in trypticase soy broth (TSB; Becton, Dickinson and Company, Sparks, MD, USA) one day before the experiments. For the microplate-based assays, bacterial suspensions were inoculated into sterile 96-well plates containing trypticase soy broth. Teicoplanin (Sanofi, İstanbul, Türkiye) was tested at final concentrations of 0.1 and 0.4 µg/mL. These concentrations were literature-based, not MIC-guided, where potential combination-associated effects of TXA (Teva, İstanbul, Türkiye) with teicoplanin could be more readily detected. TXA concentrations of 10 and 50 mg/mL were selected based on previous in vitro and implant-associated infection studies evaluating the antibacterial, antibiofilm, and antibiotic-modifying effects of TXA against staphylococci, including *S. epidermidis* [21,22,24]. In particular, 10 mg/mL TXA has been used in TXA–antibiotic combination studies involving staphylococci [22], whereas 50 mg/mL TXA has been evaluated as a higher local/topical exposure concentration in experimental biofilm and implant-associated infection models [24].

### 4.2. Disk Diffusion Assay

Disk diffusion assays were performed to evaluate the effect of teicoplanin alone or in combination with TXA on bacterial growth inhibition by using representative clinical staphylococcal isolates that were different from the isolate panel used in the microplate-based planktonic growth and crystal violet biofilm assays. Therefore, disk diffusion assays were used as supportive phenotypic observations to evaluate the apparent effect of TXA on teicoplanin-associated inhibition zones under agar-based conditions. Bacterial suspensions were adjusted to a 0.5 McFarland turbidity standard, corresponding to approximately 1.5 × 10^8^ CFU/mL, and then uniformly spread onto Mueller–Hinton agar or blood agar plates (BESLAB, Ankara, Türkiye). Antibiotic disks with a diameter of 6 mm containing 30 µg teicoplanin (Bioanalyze, ASD08700, Ankara, Türkiye) were placed onto the agar surface. For the combination groups, TXA solution was applied directly onto the teicoplanin disks after placement on the agar surface. The plates were incubated aerobically at 37 °C for approximately 18 h. After incubation, inhibition zones were evaluated and photographed for ImageJ-based quantification.

### 4.3. Quantification of Inhibition Zones

The diameters of inhibition zones were quantified from plate images using ImageJ software (version 1.54, National Institutes of Health, Bethesda, MD, USA) [30]. After incubation, agar plates were photographed using a smartphone camera under standardized conditions. Plate images were captured under the same imaging setup, with the plates placed on a flat surface and the camera positioned perpendicular to the agar surface. Images were captured under the same lighting conditions without digital zoom or image filters. The images were then imported into ImageJ, and the scale was calibrated using the known 6-mm diameter of the antibiotic disk. For each inhibition zone, the diameter was measured in millimeters by drawing a straight line across the clear zone passing through the center of the disk. When necessary, two perpendicular measurements were taken for each zone, and the mean value was used for analysis. The quantified inhibition zone diameters were then used for statistical comparison between experimental groups.

### 4.4. Measurement of Bacterial Growth

Bacterial growth was assessed using a 96-well microtiter plate assay. Fresh bacterial cultures were prepared by subculturing the isolates in trypticase soy broth one day before the experiments. Bacterial suspensions were then adjusted to a 0.5 McFarland standard and inoculated into sterile flat-bottom 96-well plates (CELLSTAR^®^, Greiner Bio-One, Kremsmünster, Austria) containing trypticase soy broth. Each well contained a final volume of 200 µL, consisting of bacterial suspension, trypticase soy broth, and the appropriate volumes of teicoplanin and/or TXA stock solutions. Teicoplanin was added to achieve final concentrations of 0.1 or 0.4 µg/mL, and TXA was added to achieve final concentrations of 10 or 50 mg/mL. The same final volume was maintained in all wells by adjusting the volume with trypticase soy broth. Control wells contained bacterial suspension and trypticase soy broth without teicoplanin or TXA. Plates were incubated for 16 h at 37 °C under static conditions in a microbiological incubator (İLDAM, Ankara, Türkiye). The following day, bacterial growth was assessed by measuring optical density at 600 nm (OD600) using a microplate reader (SpectraMax iD3, Molecular Devices, San Jose, CA, USA). All measurements were performed in technical triplicate.

### 4.5. Measurement of Biofilm Biomass Accumulation by Crystal Violet Staining

Biofilm biomass accumulation was assessed in the same 96-well microtiter plates using crystal violet staining (BESLAB, Ankara, Türkiye). After OD600 measurement, non-adherent cells were removed, and the wells were gently washed twice with 200 µL sterile distilled water to remove planktonic bacteria. The plates were then air-dried at room temperature. Attached biofilm biomass was stained with 0.1% (*w*/*v*) crystal violet solution sufficient to cover the well surface for 15 min. After staining, excess crystal violet was removed by washing the wells with distilled water, and the plates were air-dried again. No separate blank wells were included; therefore, absorbance values were analyzed relative to the untreated bacterial control within each isolate. The retained crystal violet was solubilized with 100 µL of 95% ethanol, and absorbance was measured at 570 nm (OD570) using a microplate reader (SpectraMax iD3, Molecular Devices, San Jose, CA, USA). All measurements were performed in technical triplicate.

### 4.6. Statistical Analysis

All statistical analyses were performed using GraphPad Prism version 10.0 (GraphPad Software, San Diego, CA, USA). Data are presented as mean ± standard deviation (SD) from technical triplicate measurements, unless otherwise stated. For the disk diffusion assays, inhibition zone diameters were compared between teicoplanin alone and teicoplanin plus TXA using a paired t-test for each agar condition. For planktonic growth and biofilm biomass assays, two-way analysis of variance (ANOVA) followed by Tukey’s multiple-comparisons test was used to compare treatment groups within each isolate. A *p*-value < 0.05 was considered statistically significant. As the analyses were based on technical replicates, the reported *p*-values should be interpreted as reflecting technical measurement variability within the assay rather than independent biological reproducibility. Statistical significance is indicated in the figures as follows: * *p* < 0.05; ** *p* < 0.01; *** *p* < 0.001; **** *p* < 0.0001.

### 4.7. Ethics Statement

This study was approved by the Local Institutional Ethics Committee of Ufuk University (approval no: 25.10.17.06/01).

## 5. Conclusions

In conclusion, this in vitro study shows that co-exposure to TXA and teicoplanin was associated with reduced planktonic growth, reduced biofilm biomass, and increased inhibition zone diameters under selected experimental conditions. However, because TXA-only controls were not available across the full experimental framework and formal synergy assays were not included, these findings do not establish synergistic activity or distinguish combination-specific effects from TXA-associated effects alone. Further studies including a full panel of TXA-only controls, MIC determination, checkerboard/FICI analysis, time-kill assays, and viable biofilm quantification are required to define the nature and clinical relevance of TXA–teicoplanin interactions.

## Figures and Tables

**Table 1:** Characteristics of clinical staphylococcal isolates used in the study.

Isolate ID	Assay Used	Clinical Sample/Source	Microorganism
* D1	Disk diffusion assay	Blood	Methicillin-resistant *Staphylococcus epidermidis* (MRSE)
D2	Disk diffusion assay	Blood	Methicillin-resistant *Staphylococcus epidermidis* (MRSE)
D3	Disk diffusion assay	Blood	Methicillin-resistant *Staphylococcus epidermidis* (MRSE)
D4	Disk diffusion assay	Urethral discharge	Methicillin-resistant *Staphylococcus epidermidis* (MRSE)
D5	Disk diffusion assay	Urethral discharge	*Staphylococcus epidermidis*
D6	Disk diffusion assay	Urethral discharge	*Staphylococcus epidermidis*
D7	Disk diffusion assay	Urethral discharge	*Staphylococcus epidermidis*
** G/B-1	Planktonic growth and biofilm assays	Blood	Methicillin-resistant *Staphylococcus epidermidis* (MRSE)
G/B-2	Planktonic growth and biofilm assays	Wound swab	Methicillin-resistant *Staphylococcus epidermidis* (MRSE)
G/B-3	Planktonic growth and biofilm assays	Blood	Coagulase-negative staphylococcus
G/B-4	Planktonic growth and biofilm assays	Wound swab	Coagulase-negative staphylococcus
G/B-5	Planktonic growth and biofilm assays	Urine	Coagulase-negative staphylococcus

* Isolates used for disk diffusion assays. ** Isolates used for growth and biofilm assays.

## Data Availability

The original contributions presented in this study are included in the article/Supplementary Material. Further inquiries can be directed to the corresponding author.

## Supplementary Materials

The following supporting information can be downloaded at: https://www.mdpi.com/article/10.3390/ijms27135764/s1.

## References

[1] Grupkovic J., Ceculovic M., Dabetic U., Aleksandric D., Bogosavljevic N., Lazovic R., Zagorac S. (2025). Infection in Joint Arthroplasty: Diagnosis, Prevention, and Treatment Strategies—A Comprehensive Narrative Review. Life.

[2] Flurin L., Greenwood-Quaintance K.E., Patel R. (2019). Microbiology of polymicrobial prosthetic joint infection. Diagn. Microbiol. Infect. Dis..

[3] Zimmerli W., Trampuz A., Ochsner P.E. (2004). Prosthetic-joint infections. N. Engl. J. Med..

[4] Tai D.B.G., Patel R., Abdel M.P., Berbari E.F., Tande A.J. (2022). Microbiology of hip and knee periprosthetic joint infections: A database study. Clin. Microbiol. Infect..

[5] Drago L., De Vecchi E., Bortolin M., Zagra L., Romano C.L., Cappelletti L. (2017). Epidemiology and Antibiotic Resistance of Late Prosthetic Knee and Hip Infections. J. Arthroplast..

[6] Kavolus J.J., Cunningham D.J., Rao S.R., Wellman S.S., Seyler T.M. (2019). Polymicrobial Infections in Hip Arthroplasty: Lower Treatment Success Rate, Increased Surgery, and Longer Hospitalization. J. Arthroplast..

[7] Marculescu C.E., Cantey J.R. (2008). Polymicrobial prosthetic joint infections: Risk factors and outcome. Clin. Orthop. Relat. Res..

[8] Brown M.M., Horswill A.R. (2020). *Staphylococcus epidermidis*—Skin friend or foe?. PLoS Pathog..

[9] Otto M. (2009). *Staphylococcus epidermidis*—The ‘accidental’ pathogen. Nat. Rev. Microbiol..

[10] McCann M.T., Gilmore B.F., Gorman S.P. (2008). *Staphylococcus epidermidis* device-related infections: Pathogenesis and clinical management. J. Pharm. Pharmacol..

[11] Zheng Y., Hunt R.L., Villaruz A.E., Fisher E.L., Liu R., Liu Q., Cheung G.Y.C., Li M., Otto M. (2022). Commensal *Staphylococcus epidermidis* contributes to skin barrier homeostasis by generating protective ceramides. Cell Host Microbe.

[12] Nakatsuji T., Chen T.H., Narala S., Chun K.A., Two A.M., Yun T., Shafiq F., Kotol P.F., Bouslimani A., Melnik A.V. (2017). Antimicrobials from human skin commensal bacteria protect against *Staphylococcus aureus* and are deficient in atopic dermatitis. Sci. Transl. Med..

[13] Valour F., Trouillet-Assant S., Rasigade J.P., Lustig S., Chanard E., Meugnier H., Tigaud S., Vandenesch F., Etienne J., Ferry T. (2013). Staphylococcus epidermidis in orthopedic device infections: The role of bacterial internalization in human osteoblasts and biofilm formation. PLoS ONE.

[14] Arciola C.R., Campoccia D., Speziale P., Montanaro L., Costerton J.W. (2012). Biofilm formation in Staphylococcus implant infections. A review of molecular mechanisms and implications for biofilm-resistant materials. Biomaterials.

[15] Fey P.D., Olson M.E. (2010). Current concepts in biofilm formation of *Staphylococcus epidermidis*. Future Microbiol..

[16] Buttner H., Mack D., Rohde H. (2015). Structural basis of *Staphylococcus epidermidis* biofilm formation: Mechanisms and molecular interactions. Front. Cell. Infect. Microbiol..

[17] Le K.Y., Park M.D., Otto M. (2018). Immune Evasion Mechanisms of *Staphylococcus epidermidis* Biofilm Infection. Front. Microbiol..

[18] Koch J.A., Pust T.M., Cappellini A.J., Mandell J.B., Ma D., Shah N.B., Brothers K.M., Urish K.L. (2020). *Staphylococcus epidermidis* Biofilms Have a High Tolerance to Antibiotics in Periprosthetic Joint Infection. Life.

[19] Singh R., Ray P., Das A., Sharma M. (2010). Penetration of antibiotics through *Staphylococcus aureus* and *Staphylococcus epidermidis* biofilms. J. Antimicrob. Chemother..

[20] Ker K., Edwards P., Perel P., Shakur H., Roberts I. (2012). Effect of tranexamic acid on surgical bleeding: Systematic review and cumulative meta-analysis. BMJ.

[21] Benjumea A., Diaz-Navarro M., Hafian R., Sanchez-Somolinos M., Vaquero J., Chana F., Munoz P., Guembe M. (2022). Effect of Tranexamic Acid against Staphylococcus spp. and Cutibacterium acnes Associated with Peri-Implant Infection: Results from an In Vitro Study. Microbiol. Spectr..

[22] Benjumea A., Díaz-Navarro M., Hafian R., Cercenado E., Sánchez-Somolinos M., Vaquero J., Chana F., Muñoz P., Guembe M. (2022). Tranexamic Acid in Combination with Vancomycin or Gentamicin Has a Synergistic Effect against Staphylococci. Front. Microbiol..

[23] Benjumea A., Diaz-Navarro M., Gago-Campos A.S., Visedo A., Hafian R., Cercenado E., Sanchez-Somolinos M., Munoz P., Vaquero J., Chana F. (2024). Validation of the antibacterial effect of topically applied tranexamic acid using in vitro and in vivo models. Front. Microbiol..

[24] Wang J., Zhang Z., Li J., Huang B., Jiang Z., Pan Y., He T., Hu Y., Wang L. (2022). Tranexamic acid protects against implant-associated infection by reducing biofilm formation. Sci. Rep..

[25] Fillingham Y.A., Ramkumar D.B., Jevsevar D.S., Yates A.J., Bini S.A., Clarke H.D., Schemitsch E., Johnson R.L., Memtsoudis S.G., Sayeed S.A. (2019). Tranexamic acid in total joint arthroplasty: The endorsed clinical practice guides of the American Association of Hip and Knee Surgeons, American Society of Regional Anesthesia and Pain Medicine, American Academy of Orthopaedic Surgeons, Hip Society, and Knee Society. Reg. Anesth. Pain Med..

[26] Behbahani S.B., Kiridena S.D., Wijayaratna U.N., Taylor C., Anker J.N., Tzeng T.J. (2022). pH variation in medical implant biofilms: Causes, measurements, and its implications for antibiotic resistance. Front. Microbiol..

[27] Bjarnsholt T., Alhede M., Alhede M., Eickhardt-Sorensen S.R., Moser C., Kuhl M., Jensen P.O., Hoiby N. (2013). The in vivo biofilm. Trends Microbiol..

[28] Orazi G., O’Toole G.A. (2019). “It Takes a Village”: Mechanisms Underlying Antimicrobial Recalcitrance of Polymicrobial Biofilms. J. Bacteriol..

[29] DeLeon S., Clinton A., Fowler H., Everett J., Horswill A.R., Rumbaugh K.P. (2014). Synergistic interactions of *Pseudomonas aeruginosa* and *Staphylococcus aureus* in an in vitro wound model. Infect. Immun..

[30] Schneider C.A., Rasband W.S., Eliceiri K.W. (2012). NIH Image to ImageJ: 25 years of image analysis. Nat. Methods.

