# Supplementary material for: Effects of Tranexamic Acid in Combination with Teicoplanin Against Staphylococcus isolates: Results from an In Vitro Study
Source: Int J Mol Sci. 2026 Jun 26;27(13):5764. doi: 10.3390/ijms27135764 (PMC13361542; doi:10.3390/ijms27135764)
Supplement: Supplementary file 1 [file ijms-27-05764-s001.zip › ijms-4309799-supplementary-figure.pdf]

Supplementary Figure :

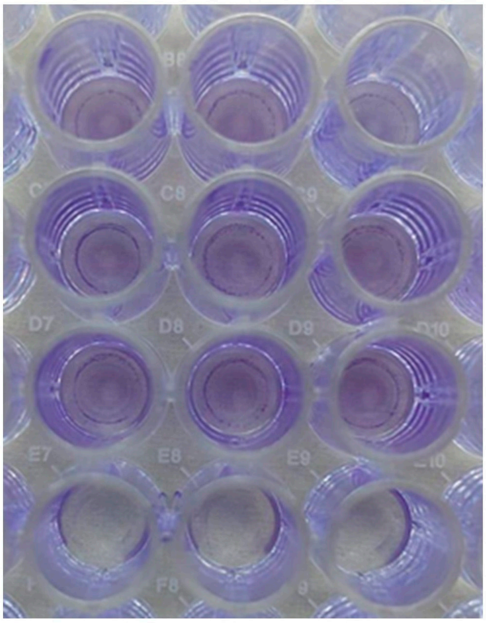

1) Isolate 3

2) Isolate 3+TEC (0.1μg/ml)

3) Isolate 3+TEC (0.1μg/ml)+ TXA 10mg/ml

4) Isolate 3+ TEC (0.1μg/ml)+ TXA 50mg/ml

Supplementary Figure S1. Representative crystal violet staining image of biofilm biomass in isolate 3 (G/B-3). Representative wells are shown for untreated control, teicoplanin (0.1 μg/mL) alone, teicoplanin (0.1 μg/mL) + TXA (10 mg/mL), and teicoplanin (0.1 μg/mL) + TXA (50 mg/mL). The image illustrates treatment-associated differences in crystal violet-stained biofilm biomass.

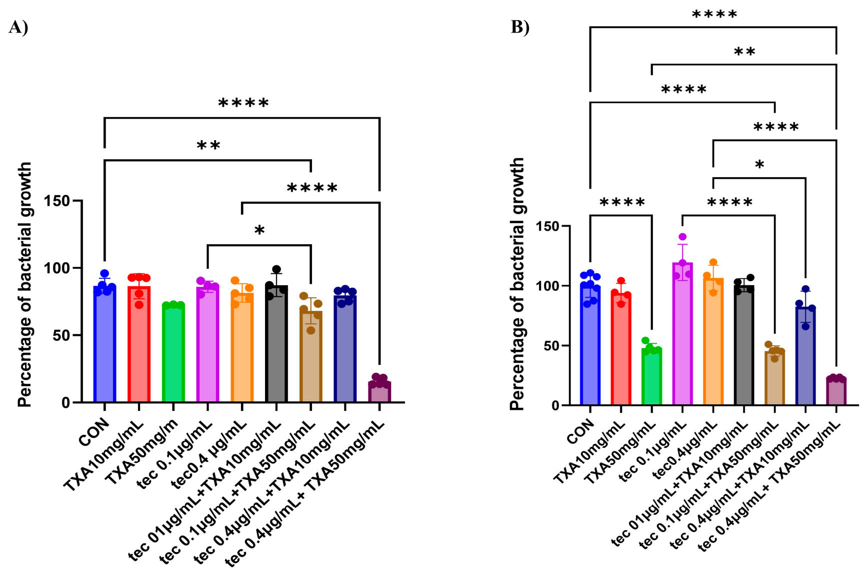

Supplementary Figure S2. Additional TXA-only control experiments in two representative isolates. (A) G/B-1 (MRSE, blood) and (B) G/B-3 (CoNS, blood) were analyzed for bacterial viability under TXA-only, teicoplanin-only, and combination treatment conditions. Data are shown as percentage of bacterial growth relative to untreated controls (mean ± SD with individual replicate values). Statistical significance was determined by two-way ANOVA followed by Tukey's multiple comparisons test. *p < 0.05, ** p < 0.01, ***p < 0.001, ****p < 0.0001.

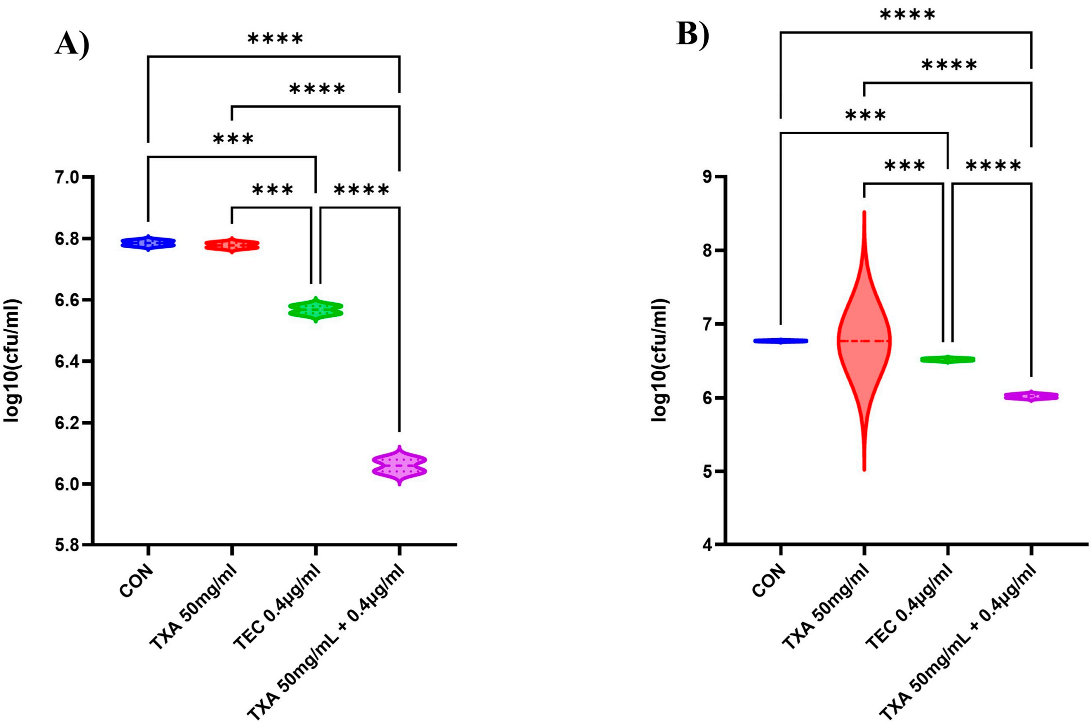

Supplementary Figure S3. CFU-based viability assay in two representative isolates under selected treatment conditions. (A) Viable counts for G/B-1 (MRSE, blood) and (B) viable counts for G/B-3 (CoNS, blood) following treatment with untreated control, TXA alone (50 mg/mL), teicoplanin alone (0.4 µg/mL), and the combination of TXA 50 mg/mL + teicoplanin 0.4 µg/mL. Data are presented as log10(CFU/mL). Statistical analysis was performed using two-way ANOVA followed by Tukey's multiple comparisons test. *$p < 0.05$, ** $p < 0.01$, ***$p < 0.001$, ****$p < 0.0001$.
