# Supplementary material for: Effects of Tranexamic Acid in Combination with Teicoplanin Against Staphylococcus isolates: Results from an In Vitro Study
Source: Int J Mol Sci. 2026 Jun 26;27(13):5764. doi: 10.3390/ijms27135764 (PMC13361542; doi:10.3390/ijms27135764)
Supplement: Supplementary file 1 [file ijms-27-05764-s001.zip › ijms-4309799-supplementary-table.pdf]

Supplementary Table S1. Mean ± SD values for planktonic bacterial growth assay.

Isolate	Treatment	Mean bacterial growth (%)	SD
Isolate 1	Control	100.00	5.55
Isolate 1	TEC 0.1 µg/mL	112.70	5.28
Isolate 1	TEC 0.1 µg/mL + TXA 10 mg/mL	118.66	6.01
Isolate 1	TEC 0.1 µg/mL + TXA 50 mg/mL	103.13	10.41
Isolate 2	Control	100.00	5.66
Isolate 2	TEC 0.1 µg/mL	101.61	3.11
Isolate 2	TEC 0.1 µg/mL + TXA 10 mg/mL	103.78	4.58
Isolate 2	TEC 0.1 µg/mL + TXA 50 mg/mL	115.30	13.17
Isolate 3	Control	100.00	13.07
Isolate 3	TEC 0.1 µg/mL	87.38	4.91
Isolate 3	TEC 0.1 µg/mL + TXA 10 mg/mL	104.21	5.39
Isolate 3	TEC 0.1 µg/mL + TXA 50 mg/mL	69.68	2.68
Isolate 4	Control	100.00	1.25
Isolate 4	TEC 0.1 µg/mL	100.68	7.11
Isolate 4	TEC 0.1 µg/mL + TXA 10 mg/mL	96.21	9.14
Isolate 4	TEC 0.1 µg/mL + TXA 50 mg/mL	83.62	5.14
Isolate 5	Control	100.00	20.18
Isolate 5	TEC 0.1 µg/mL	102.02	31.83
Isolate 5	TEC 0.1 µg/mL + TXA 10 mg/mL	74.42	16.14
Isolate 5	TEC 0.1 µg/mL + TXA 50 mg/mL	42.90	31.25

Supplementary Table S2. Mean ± SD values for planktonic bacterial growth assay with 0.4 μg/mL teicoplanin.

Isolate	Treatment	Mean bacterial growth (%)	SD
Isolate 1	Control	100.00	2.64
Isolate 1	TEC 0.4 μg/mL	80.54	9.57
Isolate 1	TEC 0.4 μg/mL + TXA 10 mg/mL	70.13	16.66
Isolate 1	TEC 0.4 μg/mL + TXA 50 mg/mL	64.71	30.73
Isolate 2	Control	100.00	1.88
Isolate 2	TEC 0.4 μg/mL	102.55	0.53
Isolate 2	TEC 0.4 μg/mL + TXA 10 mg/mL	103.16	3.13
Isolate 2	TEC 0.4 μg/mL + TXA 50 mg/mL	101.05	3.88
Isolate 3	Control	100.00	2.92
Isolate 3	TEC 0.4 μg/mL	108.08	3.19
Isolate 3	TEC 0.4 μg/mL + TXA 10 mg/mL	112.45	5.96
Isolate 3	TEC 0.4 μg/mL + TXA 50 mg/mL	62.00	0.88
Isolate 4	Control	100.00	5.51
Isolate 4	TEC 0.4 μg/mL	102.24	6.34
Isolate 4	TEC 0.4 μg/mL + TXA 10 mg/mL	101.72	15.49
Isolate 4	TEC 0.4 μg/mL + TXA 50 mg/mL	60.13	24.98
Isolate 5	Control	100.00	16.17
Isolate 5	TEC 0.4 μg/mL	87.24	11.96
Isolate 5	TEC 0.4 μg/mL + TXA 10 mg/mL	80.70	2.93
Isolate 5	TEC 0.4 μg/mL + TXA 50 mg/mL	55.59	2.77

Supplementary Table S3. Mean ± SD values for crystal violet biofilm biomass assay with 0.1 µg/mL teicoplanin.

Isolate	Treatment	Mean biofilm biomass (%)	SD
Isolate 1	Control	100.00	7.28
Isolate 1	TEC 0.1 µg/mL	132.02	13.79
Isolate 1	TEC 0.1 µg/mL + TXA 10 mg/mL	172.97	19.05
Isolate 1	TEC 0.1 µg/mL + TXA 50 mg/mL	69.35	3.33
Isolate 2	Control	100.00	12.13
Isolate 2	TEC 0.1 µg/mL	101.66	7.85
Isolate 2	TEC 0.1 µg/mL + TXA 10 mg/mL	115.02	13.13
Isolate 2	TEC 0.1 µg/mL + TXA 50 mg/mL	126.48	5.97
Isolate 3	Control	100.00	1.61
Isolate 3	TEC 0.1 µg/mL	120.48	2.61
Isolate 3	TEC 0.1 µg/mL + TXA 10 mg/mL	109.92	0.95
Isolate 3	TEC 0.1 µg/mL + TXA 50 mg/mL	41.44	3.99
Isolate 4	Control	100.00	35.58
Isolate 4	TEC 0.1 µg/mL	88.14	34.37
Isolate 4	TEC 0.1 µg/mL + TXA 10 mg/mL	57.15	21.71
Isolate 4	TEC 0.1 µg/mL + TXA 50 mg/mL	34.27	7.48
Isolate 5	Control	100.00	22.04
Isolate 5	TEC 0.1 µg/mL	89.92	10.20
Isolate 5	TEC 0.1 µg/mL + TXA 10 mg/mL	73.24	3.89
Isolate 5	TEC 0.1 µg/mL + TXA 50 mg/mL	44.84	4.97

Supplementary Table S4. Mean ± SD values for crystal violet biofilm biomass assay with 0.4 µg/mL teicoplanin.

Isolate	Treatment	Mean biofilm biomass (%)	SD
Isolate 1	Control	100.00	9.69
Isolate 1	TEC 0.4 µg/mL	108.64	3.37
Isolate 1	TEC 0.4 µg/mL + TXA 10 mg/mL	85.97	19.01
Isolate 1	TEC 0.4 µg/mL + TXA 50 mg/mL	34.04	5.20
Isolate 2	Control	100.00	4.54
Isolate 2	TEC 0.4 µg/mL	92.04	6.69
Isolate 2	TEC 0.4 µg/mL + TXA 10 mg/mL	108.70	3.25
Isolate 2	TEC 0.4 µg/mL + TXA 50 mg/mL	100.73	5.42
Isolate 3	Control	100.00	8.85
Isolate 3	TEC 0.4 µg/mL	93.82	4.82
Isolate 3	TEC 0.4 µg/mL + TXA 10 mg/mL	91.28	4.46
Isolate 3	TEC 0.4 µg/mL + TXA 50 mg/mL	37.12	7.65
Isolate 4	Control	100.00	23.39
Isolate 4	TEC 0.4 µg/mL	146.70	5.20
Isolate 4	TEC 0.4 µg/mL + TXA 10 mg/mL	106.13	17.16
Isolate 4	TEC 0.4 µg/mL + TXA 50 mg/mL	58.39	13.19
Isolate 5	Control	100.00	6.52
Isolate 5	TEC 0.4 µg/mL	104.13	7.12
Isolate 5	TEC 0.4 µg/mL + TXA 10 mg/mL	105.38	15.85
Isolate 5	TEC 0.4 µg/mL + TXA 50 mg/mL	78.66	18.31
